# Medicare Payment for Opioid Treatment Programs

**DOI:** 10.1001/jamahealthforum.2024.1907

**Published:** 2024-07-19

**Authors:** Carter H. Nakamoto, Haiden A. Huskamp, Julie M. Donohue, Michael L. Barnett, Adam J. Gordon, Ateev Mehrotra

**Affiliations:** 1 Department of Health Care Policy, Harvard Medical School, Boston, Massachusetts; 2 Department of Health Policy and Management, University of Pittsburgh School of Public Health, Pittsburgh, Pennsylvania; 3 Department of Health Policy and Management, Harvard T.H. Chan School of Public Health, Boston, Massachusetts; 4 Program for Addiction Research, Clinical Care, Knowledge, and Advocacy (PARCKA), Informatics, Decision Enhancement, and Analytic Sciences (IDEAS) Center, Department of Internal Medicine, University of Utah School of Medicine, Salt Lake City; 5 VA Salt Lake City Health Care System, Salt Lake City, Utah

## Abstract

**Importance:**

Medicare began paying for medications for opioid use disorder (MOUD) at opioid treatment programs (OTPs) that dispense methadone and other MOUD in January 2020. There has been little research describing the response to this payment change and whether it resulted in more patients receiving MOUD or just a shift in who pays for this care.

**Objective:**

To describe how many and which Medicare beneficiaries receive care from OTPs and how this compares to those receiving MOUD in other settings.

**Design, Setting, and Participants:**

This cross-sectional study included all patients receiving MOUD care identified in 2019-2022 100% US Medicare Parts B and D claims. Patients receiving care in an OTP who were dually insured with Medicare and Medicaid in the 2019-2020 Transformed Medicaid Statistical Information System were also included.

**Exposure:**

Receiving MOUD care in an OTP.

**Main Outcomes and Measures:**

Comparisons of 2022 beneficiaries treated in OTPs vs other non-OTP settings in 2022.

**Results:**

The share of Medicare beneficiaries treated by OTPs rose steadily from 4 per 10 000 (14 160 beneficiaries) in January 2020 to 7 per 10 000 (25 596 beneficiaries) in August 2020, then plateaued through December 2022; of 38 870 patients (23% ≥66 years; 35% female) treated at an OTP in 2022, 96% received methadone. Patients in OTPs, compared to those receiving MOUD in other settings, were more likely be 65 years and younger (65% vs 62%; *P* < .001), less likely to be White (72% vs 82%; *P* < .001), and more likely to be an urban resident (86% vs 74%; *P* < .001). When Medicare OTP coverage began, there was no associated drop in the number of dually insured patients with Medicaid with an OTP claim. Of the 1854 OTPs, 1115 (60%) billed Medicare in 2022, with the share billing Medicare ranging from 13% to 100% across states.

**Conclusions and Relevance:**

This study showed that since the initiation of Medicare OTP coverage in 2020, there has been a rapid increase in the number of Medicare beneficiaries with claims for OTP services for MOUD, and most OTPs have begun billing Medicare. Patients in OTPs were more likely to be urban residents and members of racial or ethnic minority groups than the patients receiving other forms of MOUD.

## Key Points

**Question:**

How many and which Medicare beneficiaries have received care from opioid treatment programs (OTPs) since Medicare began covering OTP care?

**Findings:**

In this cross-sectional study of Medicare beneficiaries from 2020 to 2022, the number of beneficiaries treated by OTPs rose steadily from 4 per 10 000 (14 160) in January 2020 to 7 per 10 000 (25 596) in August 2020, then plateaued through December 2022. Compared to those receiving medications for opioid use disorder in other settings, patients in OTPs were more likely be 65 years and younger, members of racial and ethnic minority groups, and urban residents.

**Meaning:**

There was a rapid and disproportionate increase in the number of Medicare beneficiaries with Medicare OTP claims for medications for opioid use disorder among urban residents and members of racial and ethnic minority groups.

## Teaser

This cross-sectional study describes how many and which Medicare beneficiaries receive care from opioid treatment programs and how this compares to those receiving medications for opioid use disorder in other settings.

## Introduction

Medications for opioid use disorder (MOUD)—formulations of methadone, buprenorphine, and naltrexone—are the most effective, evidence-based treatments available for OUD.^1,2,3^ However, most patients with OUD do not access these treatments. Among patients identified as having OUD in 2020, only 12.6% received MOUD in the following 6 months.^4,5^ Expanding access to MOUD is a key US policy goal.^4,6,7^

The Substance Use Disorder Prevention That Promotes Opioid Recovery and Treatment for Patients and Communities Act, passed by Congress in 2018, required Medicare to cover care at opioid treatment programs (OTPs) for the first time since the beginning of the OTP system in the 1970s.^8^ In January 2020, Medicare began paying for OTP care using weekly bundled payments in which a single payment covers management, substance use counseling, therapy, and toxicology testing, as well as MOUD, with no patient cost sharing.^9^ Because methadone can be used to treat OUD only when dispensed by certified OTPs, this also represented the first time Medicare began paying for methadone as MOUD. Prior to this change, a patient with traditional Medicare could only receive methadone treatment for OUD if they paid for the care out of pocket or, if they were dually insured with Medicaid, if the care was paid for via their Medicaid benefit.

There has been limited research describing the impact of this coverage change on receipt of care at OTPs by fee-for-service Medicare beneficiaries. Using 2020-2023 survey data from OTPs, Abraham and colleagues^10^ found that after the payment change there was rapid increase in the fraction of OTPs that accept Medicare. We build on this work using traditional fee-for-service Medicare claims, describing trends and patterns in OTP care from 2020 through 2022. We sought to understand how many patients received care at an OTP in the first 3 years after the Medicare policy change, the characteristics of patients who received MOUD care at an OTP, and what type of MOUD (methadone, buprenorphine, or naltrexone) was used. We compared patients at OTPs to those receiving MOUD care at non-OTP sites. We also characterized the share of OTPs that bill Medicare in total and by state.

We also sought to provide evidence about whether Medicare coverage of OTP care improved MOUD access and the number of patients getting care. Most Medicare beneficiaries receiving MOUD are dually insured by Medicare and Medicaid, so some of the OTP MOUD care paid for by Medicare might be the continuation of OTP care for these patients that would have been previously paid for by Medicaid. We examined this possibility by showing trends in 2019-2020 Medicaid billing for OTP care in Transformed Medicaid Statistical Information System data. Additionally, we showed 2019-2020 trends in the number of traditional Medicare beneficiaries receiving MOUD care.

## Methods

### Identifying and Categorizing Patients Receiving MOUD Care

We identified MOUD services in 2019-2022 100% fee-for-service Medicare Parts B and D claims. We identified all traditional Medicare beneficiaries (not enrolled in Medicare Advantage) receiving MOUD care at OTPs using Healthcare Common Procedure Coding System (HCPCS) codes G2067-75 (G2067, methadone treatment; G2068-72, buprenorphine treatment; and G2073-5, other MOUD treatment, including naltrexone).

Patients receiving non-OTP MOUD care were those with Part D claims for formulations of naltrexone or buprenorphine or Part B claims for clinic dispensation of these drugs (HCPCS codes J0570-5, Q9991-2, and J0592, or an HCPCS code of 96372, 11981, 11983, G0516, or G0518 with an *International Statistical Classification of Diseases and Related Health Problems, Tenth Revision,* diagnosis code beginning with F11) with any OUD diagnosis at any point in the study. We limited the study population of patients receiving non-OTP MOUD treatment to those with an OUD diagnosis (42% of all patients receiving naltrexone or buprenorphine) to distinguish MOUD from other uses of naltrexone (eg, alcohol use disorder) and buprenorphine (eg, pain management). We assumed all patients who received care at an OTP had OUD.

Finally, we identified 2019-2020 OTP billing in Medicaid among patients dually insured by Medicare and Medicaid in the Transformed Medicaid Statistical Information System data using HCPCS code H0020. Patients were not linked across the 2 datasets. Under full take-up of Medicare billing among OTPs, the share of dual-eligible beneficiaries with Medicaid OTP claims should go to zero, but we may observe residual claims due to incomplete take-up of Medicare billing or billing irregularities. Changes in the shares of dually eligible enrollees with Medicaid OTP claims around the initiation of Medicare OTP coverage allow us to quantify potential substitution between payers.

This study was approved by the Harvard Medical School institutional review board. Informed consent was not required because this study involved secondary use of administrative data. This study followed the Strengthening the Reporting of Observational Studies in Epidemiology (STROBE) reporting guideline.

### Comparison of Patient Characteristics

Beneficiary demographic information, including age, sex, and race and ethnicity, came from the Master Beneficiary Summary File. Urbanicity was determined using the US Department of Agriculture’s rural-urban commuting area codes based on beneficiary zip codes.^11^

### Estimating What Fraction of OTPs Are Billing Medicare

We used the Substance Abuse and Mental Health Services Administration online substance use and mental health treatment directory to identify all active OTPs in each state in the US at the beginning of 2022.^12^ For each state, we calculated the fraction of OTPs that billed Medicare. In the Medicare claims, we identified unique OTPs based on Carrier Claim Site of Service National Provider Identifier numbers for OTP claims. The numerator was the number of unique OTPs with at least 1 claim paid by Medicare over the period of 2020 to 2022, and the denominator was the number of OTPs in the state Substance Abuse and Mental Health Services Administration directory. In Mississippi, we capped this fraction at 100% due to the fact that several OTPs opened later in 2022 in Mississippi and billed Medicare.

Medicaid reimbursement to OTPs varies widely by state. We hypothesized that in states with low Medicaid reimbursement a larger fraction of OTPs would begin billing Medicare for beneficiaries dually eligible for both programs. We used data on each state’s Medicaid OTP payment rate according to state websites in March 2021.^13^

### Statistical Analysis

We describe 2020-2022 monthly trends in unique patients and OTPs billing traditional Medicare. In a sensitivity analysis, we compared trends in use of OTPs among patients dually insured by Medicaid vs those not dually insured. We also present 2019-2022 monthly trends of unique Medicare beneficiaries receiving any MOUD care.

Using Medicaid data, we describe 2019-2020 monthly trends of unique patients with dual Medicare and Medicaid insurance who had a Medicaid OTP claim. Using 2022 Medicare data, we compared several patient characteristics (Medicaid eligibility, age [≤40, 41-65, or ≥66 years], race and ethnicity [Black, Hispanic, or White], sex, and urban residence [rural-urban commuting area ≤3]) for patients who had any methadone use for OUD at OTPs vs those who only received other MOUD at OTPs in 2022. We conducted an analogous comparison with the same characteristics for those who had any OTP care vs those who only received MOUD care elsewhere in non-OTP settings (who had an OUD diagnosis). Because we wanted to ensure that we could capture an OUD diagnosis, we limited the cohort in this second analysis to those with 12 months of Medicare Part B coverage in the calendar year and no months of Medicare Advantage coverage. We used χ^2^ tests to compare categorical variables. Two-sided *P* < .05 indicated statistical significance, and analyses were conducted using SAS, version 9.4M7 (SAS Institute).

## Results

### Patient Characteristics

**Figure 1.**
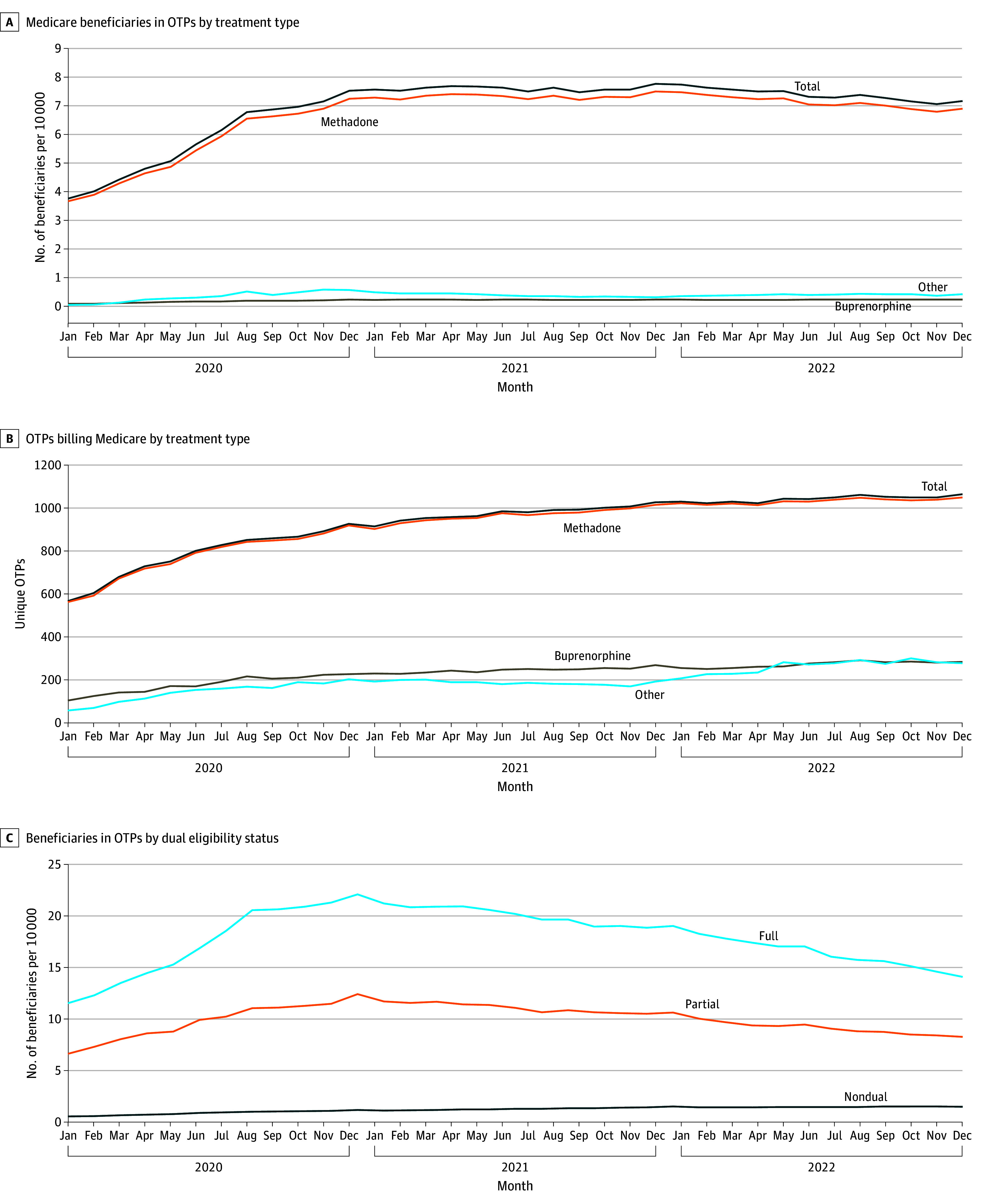
Trends in Opioid Treatment Program (OTP) Billing in Fee-for-Service Medicare, 2020-2022 Other includes naltrexone and unspecified medications for the treatment of opioid use disorder.

The share of beneficiaries receiving OTP care per month rose from 4 per 10 000 (14 160 beneficiaries) in January 2020 to 7 per 10 000 (25 596 beneficiaries) in August 2020 (Figure 1A), then plateaued. Across the 38 870 patients who received care at an OTP at any point in 2022, 23% were 66 years and older, 66% had a disability, and 66% were dually eligible for both Medicare and Medicaid insurance (Table 1).

**Table 1.** Characteristics of Medicare Beneficiaries Receiving Care at Opioid Treatment Programs (OTPs) in 2022

Characteristic	Beneficiaries, %			*P* value
	Any OTP care	Type of MOUD received		
		Any methadone	Only MOUD other than methadone	
Total No.	38 870	37 140	1730	NA
Dual eligible	66	66	58	<.001
With a disability	66	66	72	<.001
Age, y				
≤40	28	28	36	<.001
41-65	49	49	49	.77
≥66	23	23	15	<.001
Sex				
Female	35	35	36	.10
Male	65	65	64	
Race and ethnicity^a^				
Black	14	14	9	<.001
Hispanic	10	10	5	<.001
White	59	59	67	<.001
Urban resident	78	78	84	<.001
US region				
West	21	21	14	<.001
Midwest	12	11	29	<.001
Northeast	27	28	10	<.001
South	26	26	31	<.001

Abbreviations: MOUD, medication for opioid use disorder; NA, not applicable.

^a^ The race and ethnicity variable comes from the Beneficiary Race Code from the Master Beneficiary Summary File for the Medicare claims data. Race and ethnicity data are displayed to capture potential disparities across groups in receipt of medications for opioid use disorder. Other race and ethnicity categories are excluded due to small numbers.

### Patients Receiving OTP Care Paid for Via Medicare and Patients Receiving Methadone vs Other Forms of MOUD

Among the 38 870 patients in the Medicare population who received care at an OTP at any point in 2022, 96% received any methadone treatment, with the remainder largely receiving buprenorphine or naltrexone (Table 1). Patients in OTPs who received any methadone, compared to those who only received a different MOUD, were more likely to be dually insured in Medicaid (66% vs 58%; *P* < .001), less likely to be White (59% vs 67%; *P* < .001), and more likely to be located in the West (21% vs 14%; *P* < .001).

### Patients Receiving MOUD at an OTP vs MOUD Care in Other Settings

**Figure 2.**
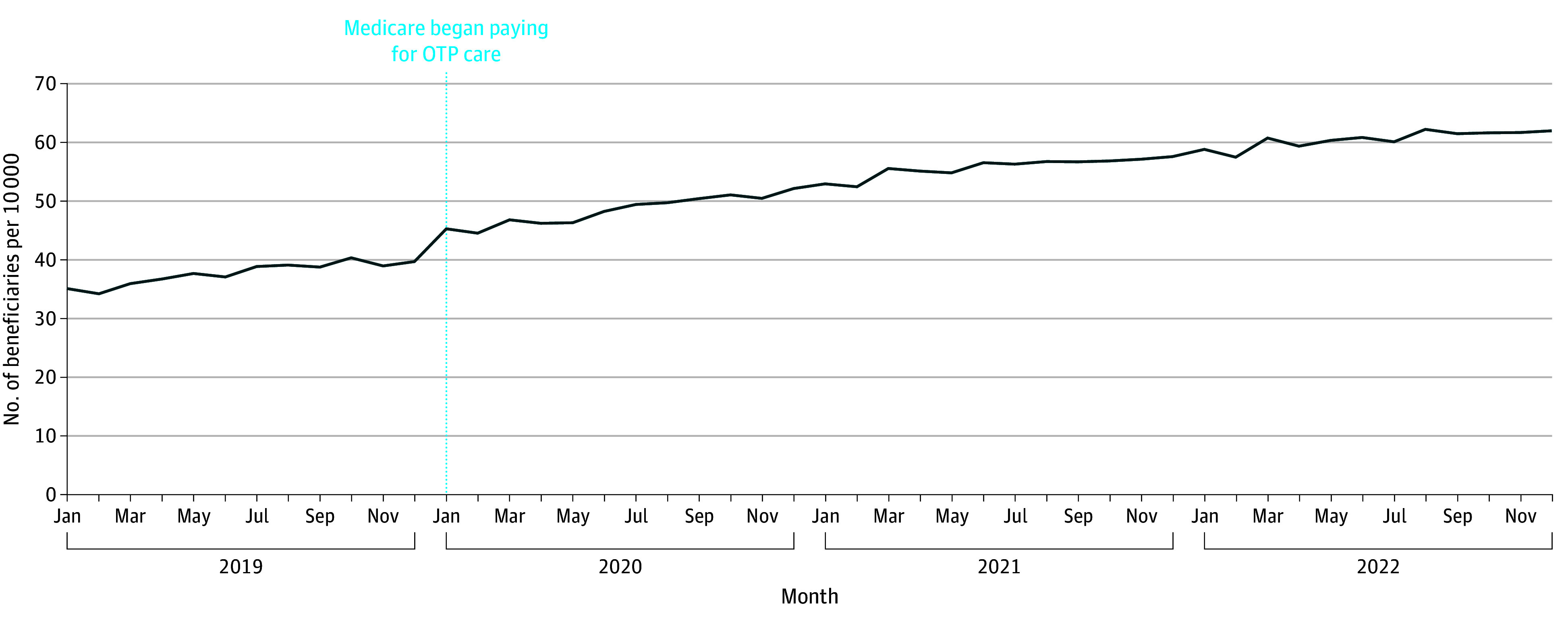
Medicare Beneficiaries Receiving Any Form of Medication for Opioid Use Disorder at Opioid Treatment Programs (OTPs) or Other Outpatient Settings

Among all fee-for-service Medicare beneficiaries, the share of patients receiving MOUD in any setting steadily rose from 35 per 10 000 (134 604 beneficiaries) in January 2019 to 62 per 10 000 (219 688 beneficiaries) in December 2022 for an average monthly increase of 0.6 per 10 000 (1811 beneficiaries) (Figure 2). In contrast, between December 2019 and January 2020 (the month that Medicare payment of OTP care began), the number of patients receiving any MOUD increased by 5 per 10 000 (16 253 patients)—an order of magnitude more.

In 2022, after limiting to enrollees with a full year of fee-for-service coverage (ie, no Medicare Advantage coverage) there were 66 247 patients receiving MOUD reimbursed by Medicare at a non-OTP care setting and 25 296 patients receiving care at an OTP (Table 2). Patients seen at OTPs, compared to those getting MOUD in non-OTP outpatient settings, were less likely to be 66 years or older (35% vs 38%; *P* < .001), less likely to be White (72% vs 82%; *P* < .001), more likely to be urban residents (86% vs 74%; *P* < .001), and more likely to live in the Northeast (30% vs 25%; *P* < .001).

**Table 2.** Characteristics of Medicare Beneficiaries Receiving Medication for Opioid Use Disorder at Opioid Treatment Programs (OTPs) vs Other Outpatient Settings in 2022^a^

Characteristic	Beneficiaries, %		*P* value
	OTP	Other setting	
Total No.	25 296	66 247	NA
Dual eligible	78	65	<.001
With a disability	74	75	.002
Age, y			
≤40	14	13	.14
41-65	52	49	<.001
≥66	35	38	<.001
Sex			
Female	41	51	<.001
Male	59	49	<.001
Race and ethnicity^b^			
Black	14	7	<.001
Hispanic	10	6	<.001
White	72	82	<.001
Urban resident	86	74	<.001
US region			
West	25	25	.05
Midwest	13	17	<.001
Northeast	30	25	<.001
South	31	33	<.001

Abbreviation: NA, not applicable.

^a^ Totals for OTP users are smaller than in Table 1 because the population was limited to those with 12 months of Medicare Part B coverage in 2022, with no enrollment in Medicare Advantage.

^b^ The race and ethnicity variable comes from the Beneficiary Race Code from the Master Beneficiary Summary File for the Medicare claims data. Race and ethnicity data are displayed to capture potential disparities across groups in receipt of medications for opioid use disorder. Other race and ethnicity categories are excluded due to small numbers.

### Payment for OTP Among Patients Dually Insured by Medicare and Medicaid

**Figure 3.**
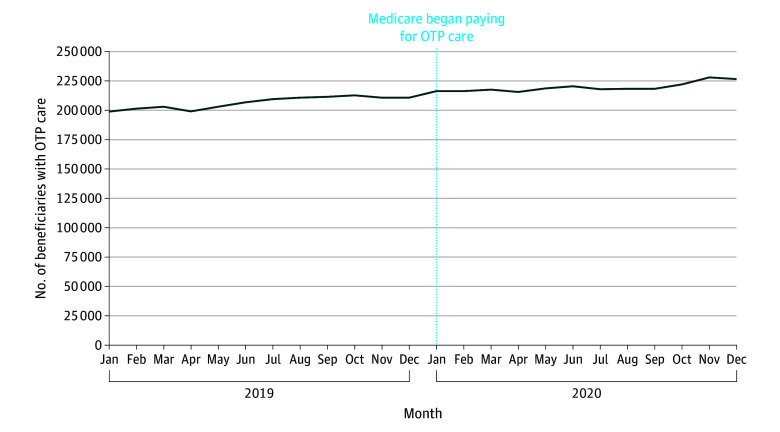
Patients Dually Insured by Medicare and Medicaid With Opioid Treatment Program (OTP) Care Paid for Via Medicaid

Among the Medicare population who had full dual coverage with Medicaid, there was a steady increase throughout 2020 in the share with OTP claims from 12 per 10 000 in January to 22 per 10 000 in December, followed by smaller steady decreases through 2022 (Figure 1C). In national Medicaid data, the share of dually eligible beneficiaries receiving methadone treatment rose from January 2019 to December 2020 (Figure 3). There was no drop in the share of dually eligible beneficiaries with Medicaid methadone claims from December 2019 (269 per 10 000 [210 660 patients]) to January 2020 (276 per 10 000 [216 307 patients]) when Medicare began paying for methadone treatment.

### Fraction of OTPs Billing Medicare

The number of OTPs billing Medicare rose from 567 in January 2020 to 1065 in December 2022 (Figure 1B). There were 1854 OTPs in the US at the beginning of 2022, and 1115 (60%) billed Medicare from 2020 through 2022. This fraction ranged from 13% to 100% across states (eFigure 1 in Supplement 1). There was no clear association between the state’s Medicaid payment rates and what fraction of OTPs billed Medicare (eFigure 2 in Supplement 1).

### State-Level Variation in OTP Services

At the state level, OTP use per capita among Medicare beneficiaries was not associated with the share of OTPs that bill Medicare (eFigure 3 in Supplement 1) but appears associated with the number of OTPs per capita (eFigure 4 in Supplement 1) and the number of OTPs billing Medicare per capita (eFigure 5 in Supplement 1).

## Discussion

After the introduction of Medicare payment for OTP care in January 2020, there was rapid uptake of OTP care reimbursed by Medicare. In 2022, 38 870 patients received care at an OTP billed by Medicare.

These results provide suggestive evidence that the initiation of Medicare coverage of OTP care led to more Medicare beneficiaries receiving MOUD. After the start of Medicare’s payment policy, we observed that the total share of beneficiaries receiving MOUD (OTP or non-OTP) nearly doubled without evidence of a switch in payer from Medicaid to Medicare. There are several important caveats. About 6% of patients pay cash for OTP care, and a minority of OTPs only accept cash payments.^14,15^ It is possible that some Medicare beneficiaries were paying cash for their OTP care prior to 2020, then after January 2020 Medicare paid for their OTP care instead. To the degree that this is the case, this would not represent an increase in the number of patients receiving MOUD and instead mean that insurance was now paying for the care. It is also important to acknowledge that, at least among the Medicare fee-for-service population, most of the growth in MOUD treatment from 2020 to 2022 comes from greater use of MOUD care outside an OTP. A relative minority of patients are getting care at an OTP.

While the share of full dual beneficiaries receiving methadone treatment was higher at the end of 2022 than at the beginning of 2020, this share decreased in 2021 to 2022. This merits further investigation; recent qualitative work has found that some patients with OUD who use fentanyl are fearful of starting methadone due to risks of precipitated withdrawal.^16^ This is one potential driver of this finding.

Patients receiving Medicare-financed OTP care, who mostly receive methadone treatment, are largely adults younger than 65 years with a disability. Compared to patients receiving MOUD in other settings, who mostly receive buprenorphine treatment, patients receiving MOUD at an OTP were more likely to be urban residents and Black or Hispanic. These findings echo the experience among Medicare Advantage enrollees in 1 national plan.^6^ Consistent with prior survey work, we found that the majority of OTPs were billing Medicare by the end of 2022.^10,17^

In the past 5 years, there has been a dramatic increase in the number of OTPs in the US, and Medicare’s new coverage for OTP care could be one contributing factor to this growth.^18^ This is meaningful, as we observed associations between the number of OTPs and the share of Medicare beneficiaries accessing OTP services at the state level. However, it is unclear why a substantial minority of OTPs were not billing Medicare. One potential explanation is that some OTPs lack the administrative structure to bill insurers and, therefore, are unlikely to enroll as a Medicare provider and bill Medicare for care. Another open question is why the fraction of OTPs billing Medicare varied substantially by state. While we did not find an association between state Medicaid payment rates and what fraction of OTPs in the state are billing Medicare, there is substantial variation in state regulation of OTPs (eg, allowing take-home doses), and these other regulations could have influenced whether an OTP chooses to bill Medicare.^19,20^

### Limitations

There were several key limitations of this study. First, claims data do not capture many details of the care provided by OTPs. For example, we do not know how many therapy services were provided by the OTP as part of Medicare’s OTP bundled payments. Second, the COVID-19 pandemic occurred soon after the beginning of Medicare payment for OTPs, and it is difficult to disentangle its impact on MOUD care from that of Medicare’s OTP coverage policy. Third, in the Medicaid analysis, we measured methadone treatment using a single treatment code (HCPCS code H0020). We acknowledge that there is variation across state Medicaid programs in how OTPs bill for payment, and it is possible that there were changes in those billing rules at the start of 2020. Fourth, in the analysis of non-OTP–based MOUD treatment, it was unclear how to distinguish between those receiving buprenorphine or naltrexone for treatment of OUD vs other reasons. Focusing only on those with an OUD diagnosis is likely overly restrictive given undercoding of OUD in claims; however, including all patients receiving these medications would have yielded an overestimate, and MOUD use cases cannot be cleanly separated from pain management (eg, buprenorphine formulations) or alcohol use disorder (eg, naltrexone formulations) use cases by focusing only on formulations commonly used for MOUD or other variables available in claims data.^21,22^

## Conclusions

This cross-sectional study showed that since the initiation of Medicare OTP coverage in 2020, there has been a rapid increase in the number of Medicare beneficiaries with claims for OTP services for MOUD, and most OTPs have begun billing Medicare. Patients at OTPs are more likely to be urban residents and members of racial or ethnic minority groups than the patients receiving other forms of MOUD.

## Notes

Supplement 1. **eFigure 1.** Fraction of OTPs in each state that are billing Medicare **eFigure 2. **Mean State-Level Fraction of OTPs that are billing Medicare by Medicaid:Medicare Fee Ratio **eFigure 3. **Number of Medicare enrollees using OTPs per 10,000 enrollees vs. Share of OTPs billing Medicare by State, 2022 **eFigure 4. **Number of Medicare enrollees using OTPs per 10,000 enrollees vs. OTPs per million people by State, 2022 **eFigure 5. **Number of Medicare enrollees using OTPs per 10,000 enrollees vs. OTPs billing Medicare per million people by State, 2022

Supplement 2. Data Sharing Statement
