# Supplementary material for: Medicare Payment for Opioid Treatment Programs
Source: JAMA Health Forum. 2024 Jul 19;5(7):e241907. doi: 10.1001/jamahealthforum.2024.1907 (PMC11259898; doi:10.1001/jamahealthforum.2024.1907)
Supplement: Supplement 1. — eFigure 1. Fraction of OTPs in each state that are billing Medicare eFigure 2. Mean State-Level Fraction of OTPs that are billing Medicare by Medicaid:Medicare Fee Ratio eFigure 3. Number of Medicare enrollees using OTPs per 10,000 enrollees vs. Share of OTPs billing Medicare by State, 2022 eFigure 4. Number of Medicare enrollees using OTPs per 10,000 enrollees vs. OTPs per million people by State, 2022 eFigure 5. Number of Medicare enrollees using OTPs per 10,000 enrollees vs. OTPs billing Medicare per million people by State, 2022 [file jamahealthforum-e241907-s001.pdf]

Supplemental Online Content

Nakamoto CH, Huskamp HA, Donohue JM, Barnett ML, Gordon AJ, Mehrotra A. Medicare payment for opioid treatment programs. *JAMA Health Forum*. 2024;5(7):e241907. doi:10.1001/jamahealthforum.2024.1907

eFigure 1. Fraction of OTPs in each state that are billing Medicare
eFigure 2. Mean State-Level Fraction of OTPs that are billing Medicare by Medicaid:Medicare Fee Ratio
eFigure 3. Number of Medicare enrollees using OTPs per 10,000 enrollees vs. Share of OTPs billing Medicare by State, 2022
eFigure 4. Number of Medicare enrollees using OTPs per 10,000 enrollees vs. OTPs per million people by State, 2022
eFigure 5. Number of Medicare enrollees using OTPs per 10,000 enrollees vs. OTPs billing Medicare per million people by State, 2022

This supplemental material has been provided by the authors to give readers additional information about their work.

© 2024 Nakamoto CH et al. *JAMA Health Forum.*

Figure S1. Fraction of OTPs in each state that are billing Medicare

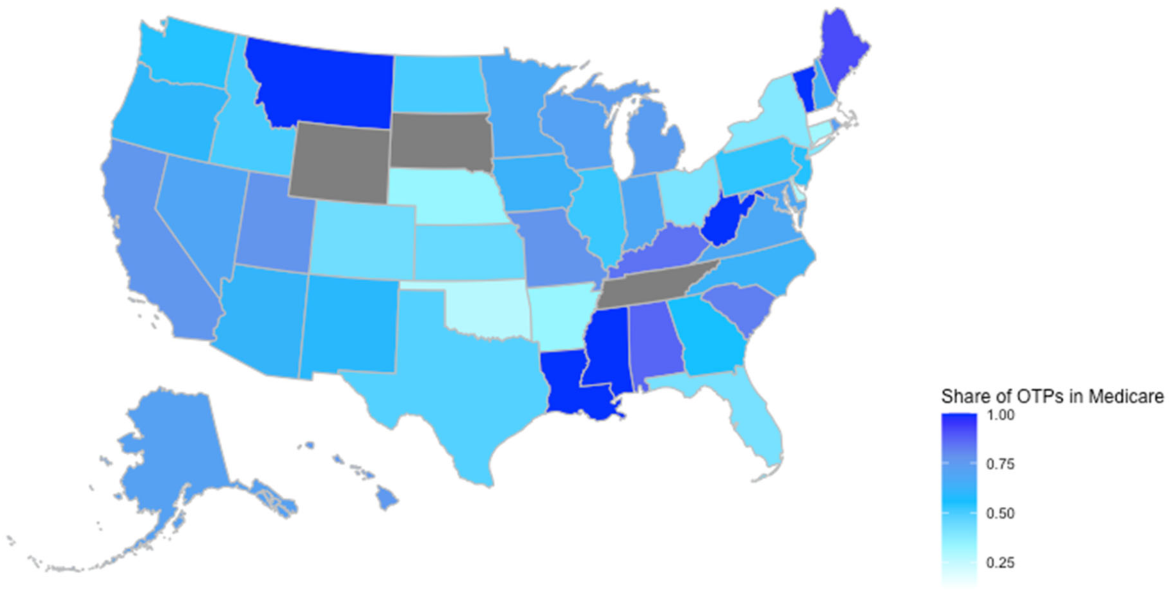

1. Mississippi has 6 OTPs that bill Medicare but 4 OTPs in the SAMHSA registry. This appears to be due to some erroneous Medicare billing and a lag in SAMHSA registry updates. It has been graphed as 100%.
2. South Dakota, Tennessee, and Wyoming are grey to reflect missing data: South Dakota and Tennessee have no OTPs billing Medicare fee-for-service, and Wyoming has no OTPs.

© 2024 Nakamoto CH et al. *JAMA Health Forum.*

Figure S2: Mean State-Level Fraction of OTPs that are billing Medicare by Medicaid:Medicare Fee Ratio

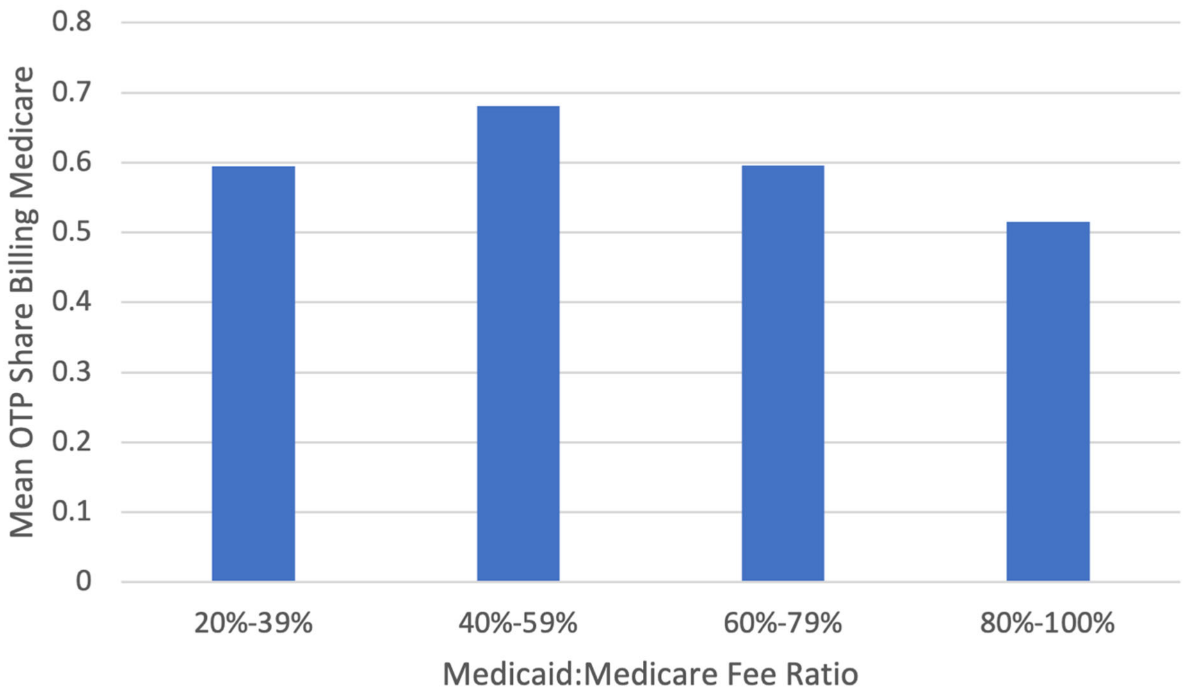

* Medicaid:Medicare fee ratios calculated based on state website fee reports in May 2021, taken from Clemans-Cope et al. (2022).

© 2024 Nakamoto CH et al. *JAMA Health Forum.*

Figure S3: Number of Medicare enrollees using OTPs per 10,000 enrollees vs. Share of OTPs billing Medicare by State, 2022

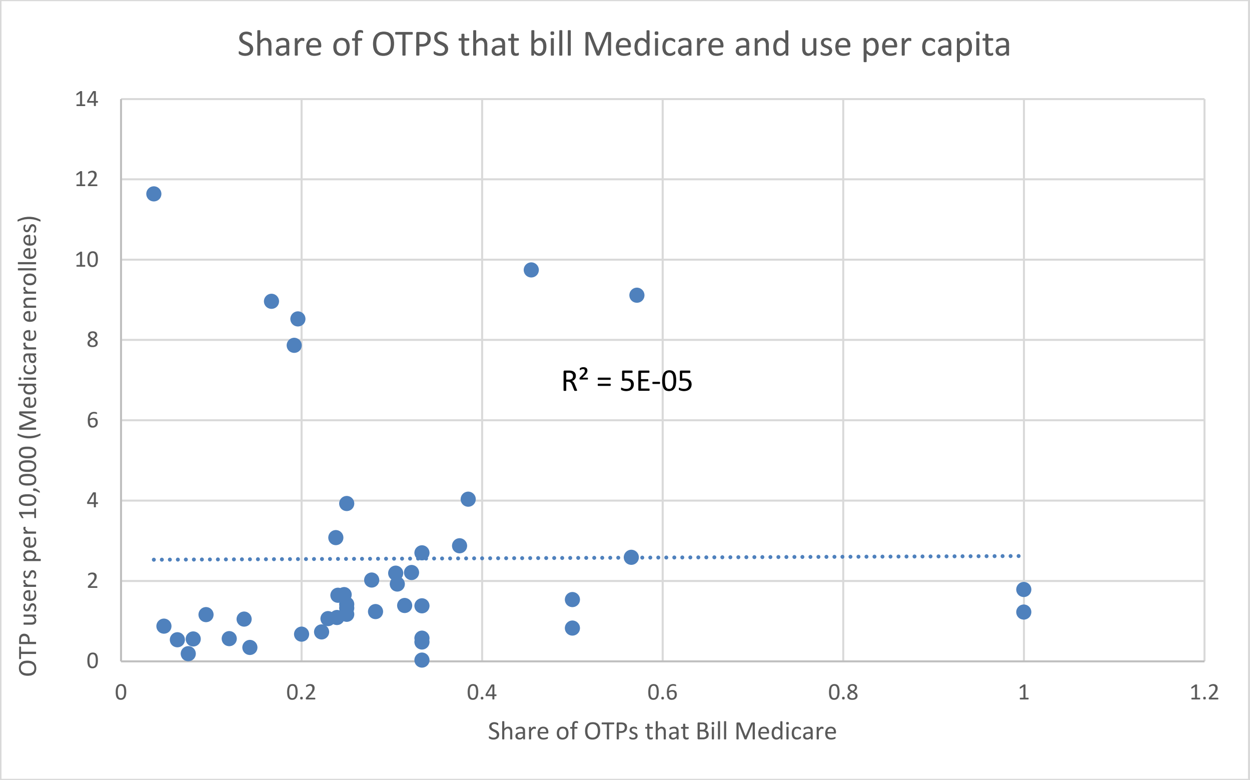

Mississippi has 6 OTPs that bill Medicare but 4 OTPs in the SAMHSA registry. This appears to be due to some erroneous Medicare billing and a lag in SAMHSA registry updates. It has been graphed as 100%.

© 2024 Nakamoto CH et al. *JAMA Health Forum.*

Figure S4: Number of Medicare enrollees using OTPs per 10,000 enrollees vs. OTPs per million people by State, 2022

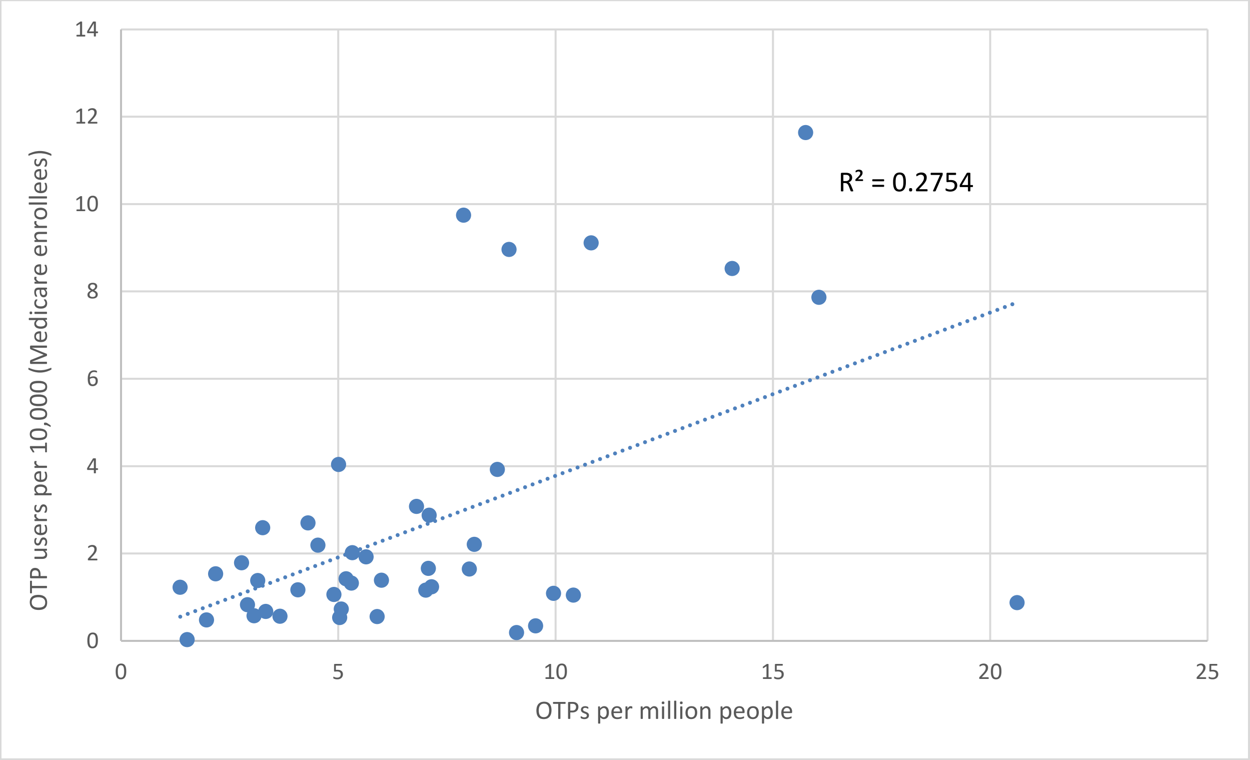

© 2024 Nakamoto CH et al. *JAMA Health Forum.*

Figure S5: Number of Medicare enrollees using OTPs per 10,000 enrollees vs. OTPs billing Medicare per million people by State, 2022

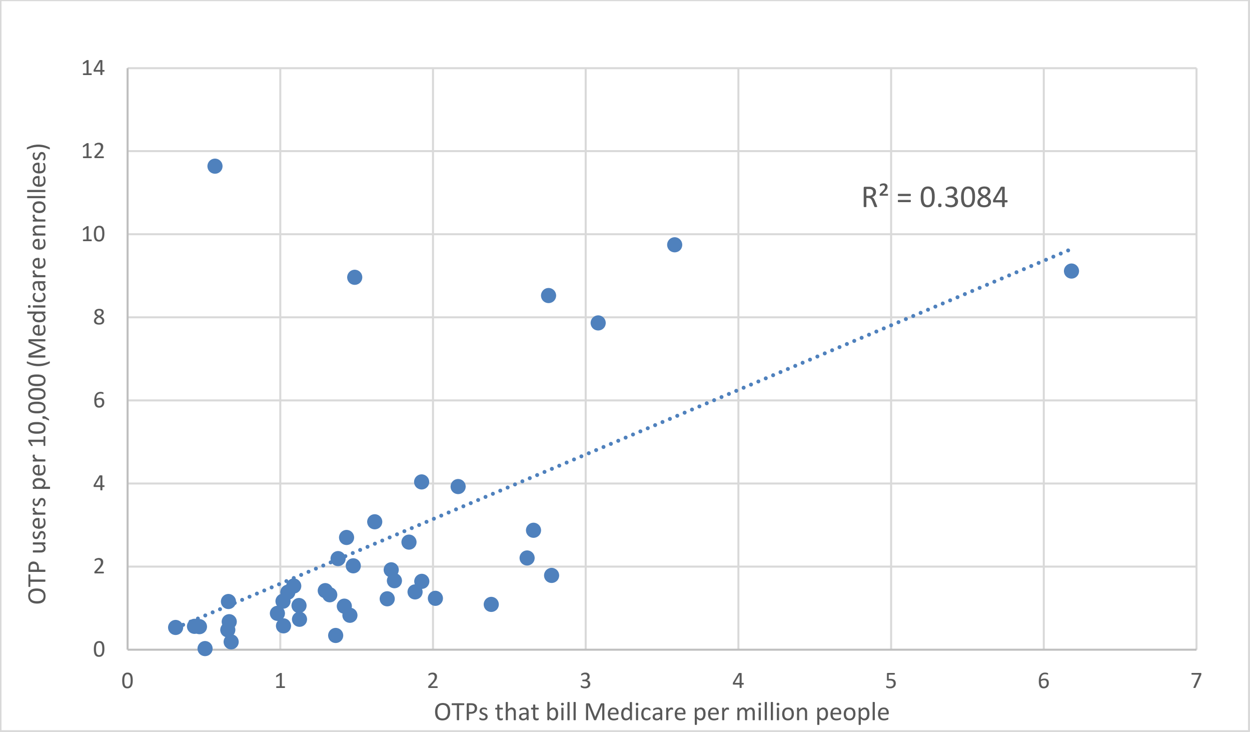

© 2024 Nakamoto CH et al. *JAMA Health Forum.*
