# Supplementary material for: Medicare Payment for Opioid Treatment Programs
Source: JAMA Health Forum. 2024 Jul 19;5(7):e241907. doi: 10.1001/jamahealthforum.2024.1907 (PMC11259898; doi:10.1001/jamahealthforum.2024.1907)
Supplement: Supplement 2. — Data Sharing Statement [file jamahealthforum-e241907-s002.pdf]

## Data Sharing Statement

Nakamoto. Medicare Payment for Opioid Treatment Programs. *JAMA Health Forum*. Published July 19, 2024. doi:10.1001/jamahealthforum.2024.1907

**Data**
**Data available:** No

**Additional Information**
**Explanation for why data not available:** Due to the terms of our data use agreement with the Centers for Medicare & Medicaid Services, we are not able to share the insurance claims data.
